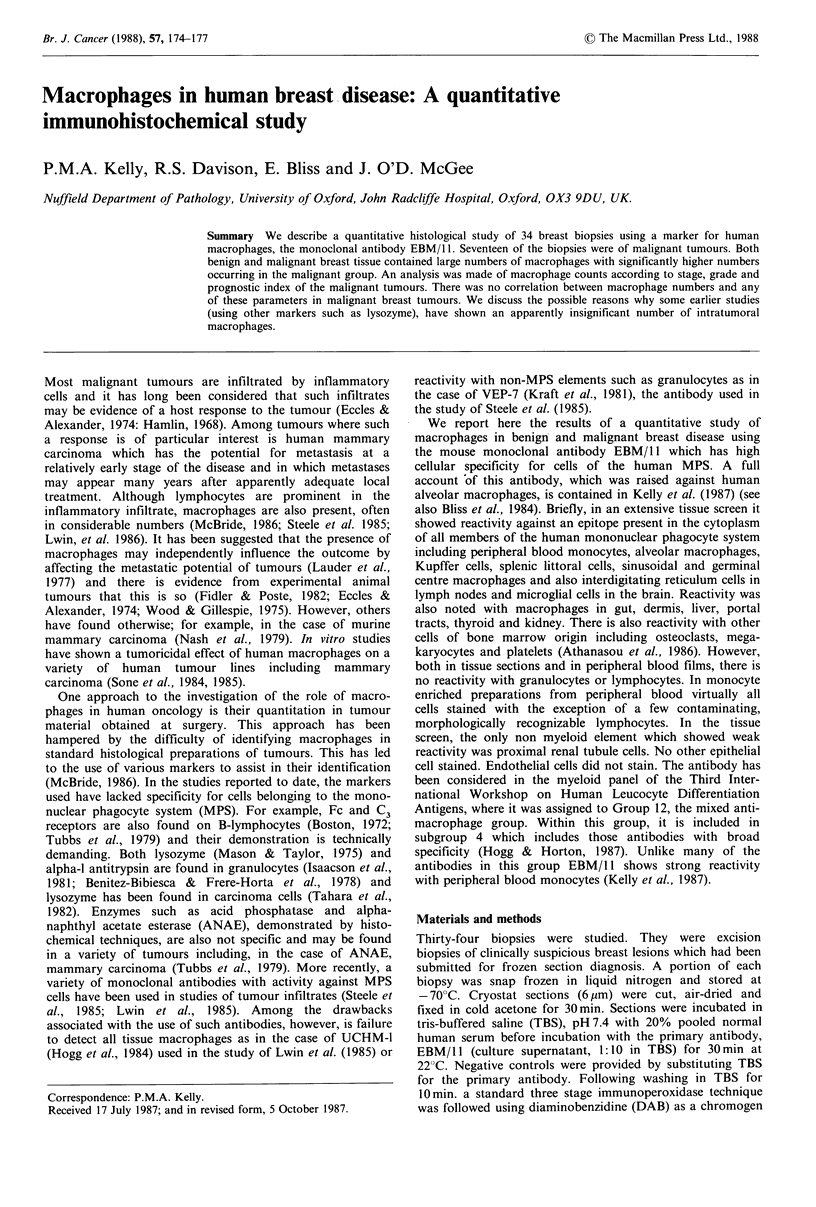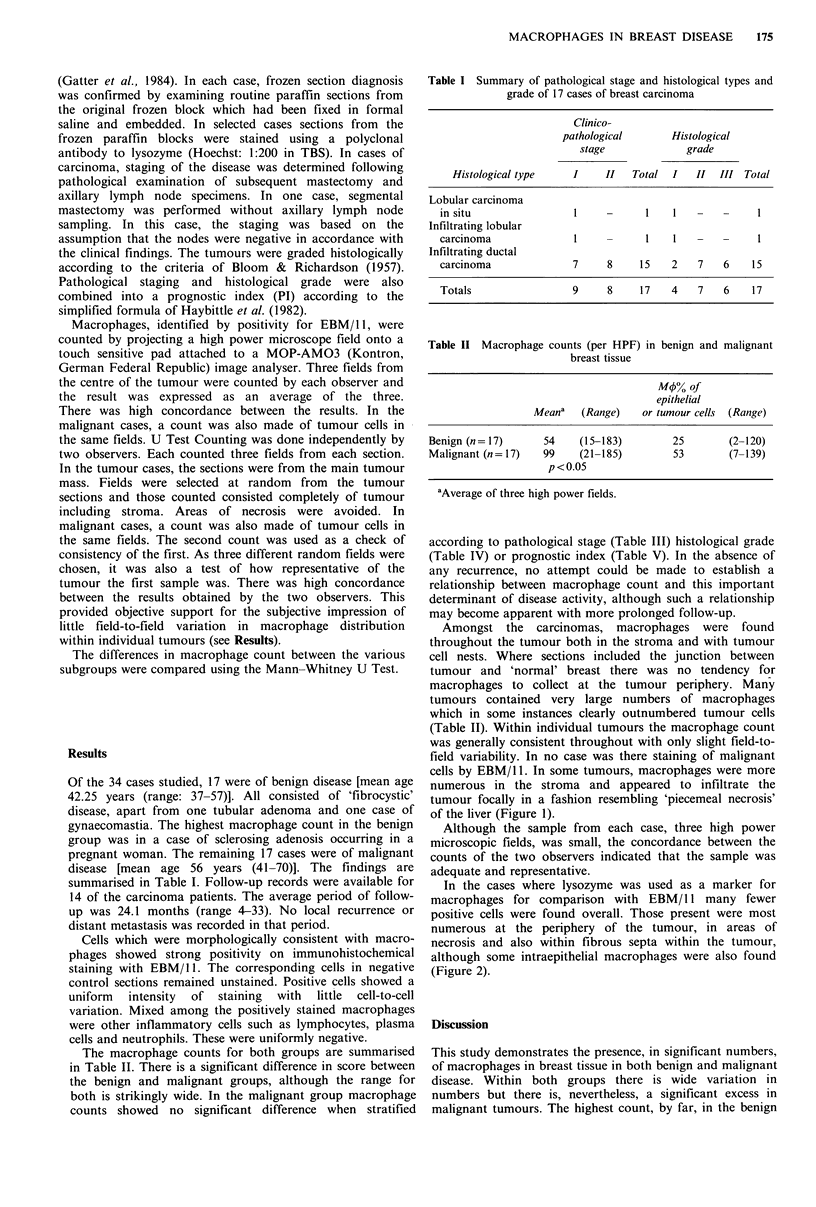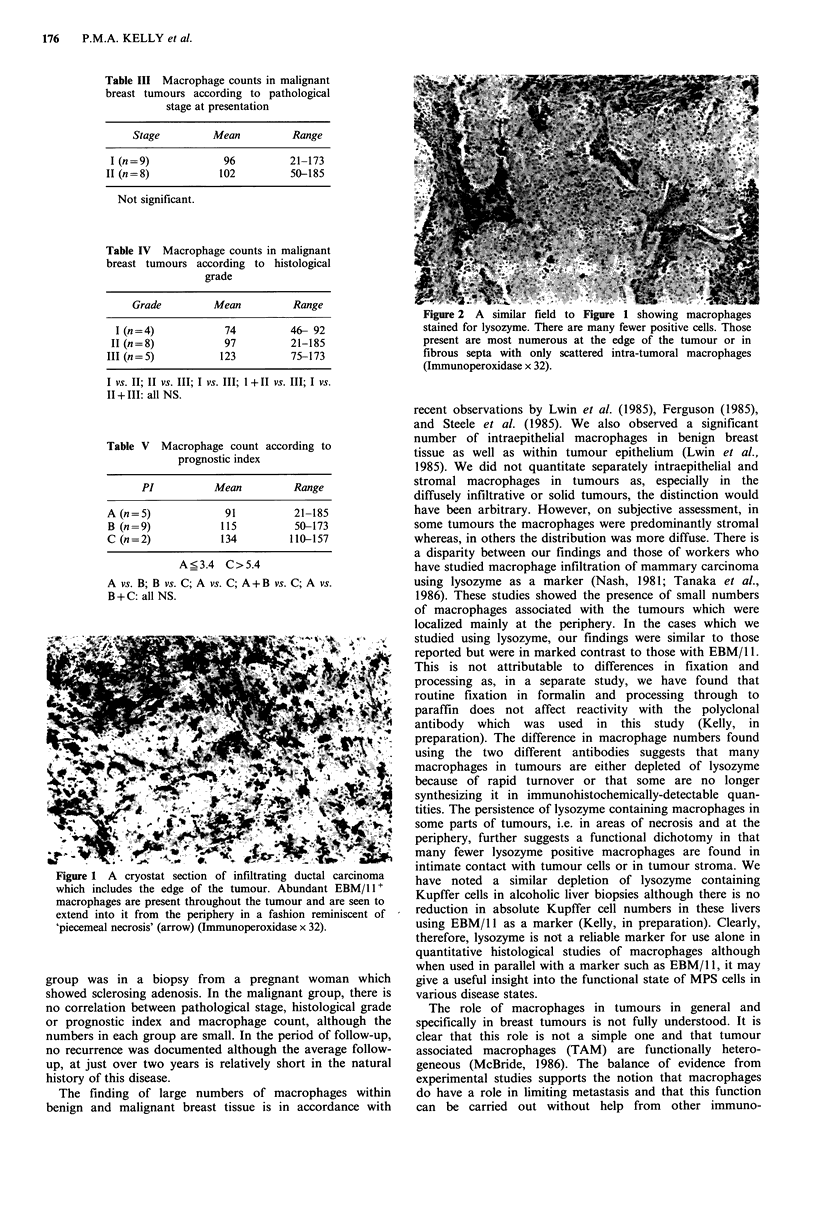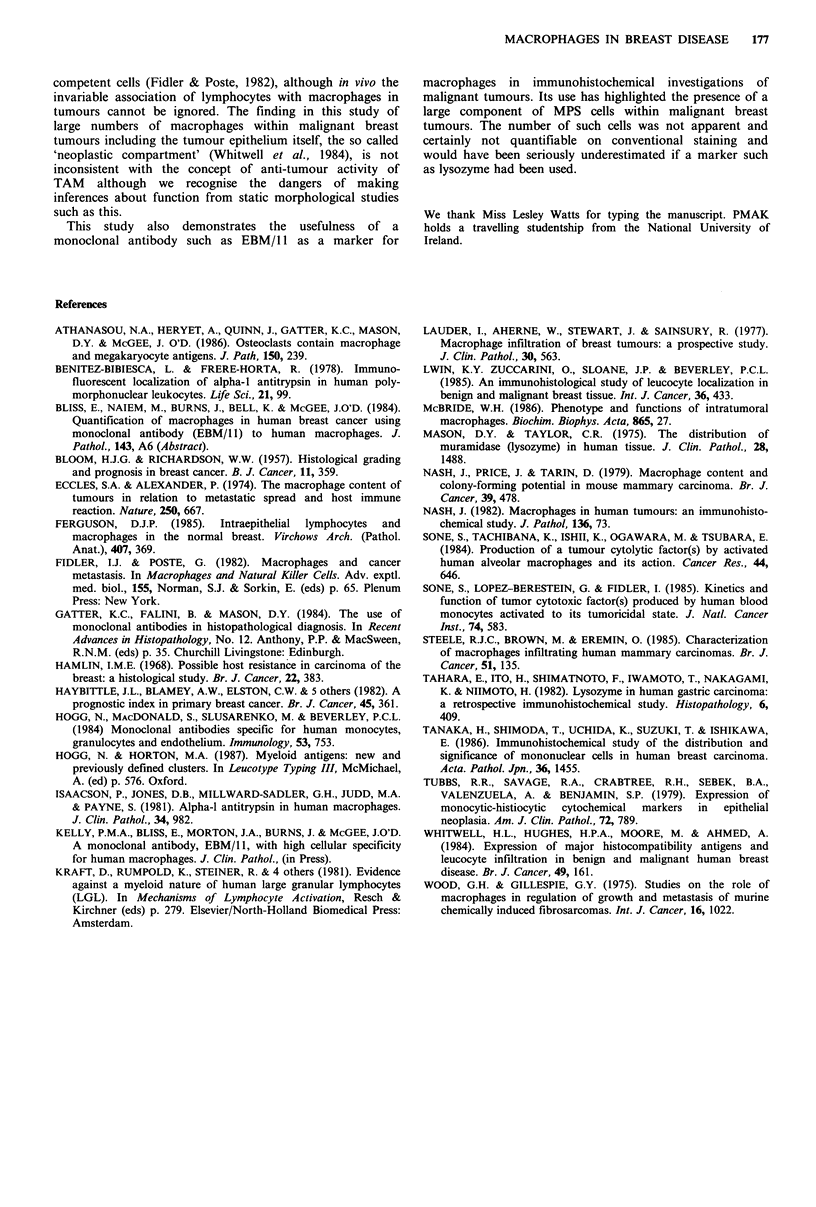# Macrophages in human breast disease: a quantitative immunohistochemical study.

**DOI:** 10.1038/bjc.1988.36

**Published:** 1988-02

**Authors:** P. M. Kelly, R. S. Davison, E. Bliss, J. O. McGee

**Affiliations:** Nuffield Department of Pathology, University of Oxford, John Radcliffe Hospital, UK.

## Abstract

**Images:**


*Br. J. Cancer* (1988), **57**, 174–177 © The Macmillan Press Ltd., 1988

### Macrophages in human breast disease: A quantitative immunohistochemical study

P.M.A. Kelly, R.S. Davison, E. Bliss and J. O'D. McGee

*Nuffield Department of Pathology, University of Oxford, John Radcliffe Hospital, Oxford, OX3 9DU, UK.*

**Summary** We describe a quantitative histological study of 34 breast biopsies using a marker for human macrophages, the monoclonal antibody EBM/11. Seventeen of the biopsies were of malignant tumours. Both benign and malignant breast tissue contained large numbers of macrophages with significantly higher numbers occurring in the malignant group. An analysis was made of macrophage counts according to stage, grade and prognostic index of the malignant tumours. There was no correlation between macrophage numbers and any of these parameters in malignant breast tumours. We discuss the possible reasons why some earlier studies (using other markers such as lysozyme), have shown an apparently insignificant number of intratumoral macrophages.

Most malignant tumours are infiltrated by inflammatory cells and it has long been considered that such infiltrates may be evidence of a host response to the tumour (Eccles & Alexander, 1974: Hamlin, 1968). Among tumours where such a response is of particular interest is human mammary carcinoma which has the potential for metastasis at a relatively early stage of the disease and in which metastases may appear many years after apparently adequate local treatment. Although lymphocytes are prominent in the inflammatory infiltrate, macrophages are also present, often in considerable numbers (McBride, 1986; Steele *et al.* 1985; Lwin, *et al.* 1986). It has been suggested that the presence of macrophages may independently influence the outcome by affecting the metastatic potential of tumours (Lauder *et al.*, 1977) and there is evidence from experimental animal tumours that this is so (Fidler & Poste, 1982; Eccles & Alexander, 1974; Wood & Gillespie, 1975). However, others have found otherwise; for example, in the case of murine mammary carcinoma (Nash *et al.*, 1979). *In vitro* studies have shown a tumoricidal effect of human macrophages on a variety of human tumour lines including mammary carcinoma (Sone *et al.*, 1984, 1985).

One approach to the investigation of the role of macrophages in human oncology is their quantitation in tumour material obtained at surgery. This approach has been hampered by the difficulty of identifying macrophages in standard histological preparations of tumours. This has led to the use of various markers to assist in their identification (McBride, 1986). In the studies reported to date, the markers used have lacked specificity for cells belonging to the mononuclear phagocyte system (MPS). For example, Fc and $C_3$ receptors are also found on B-lymphocytes (Boston, 1972; Tubbs *et al.*, 1979) and their demonstration is technically demanding. Both lysozyme (Mason & Taylor, 1975) and alpha-l antitrypsin are found in granulocytes (Isaacson *et al.*, 1981; Benitez-Bibiesca & Frere-Horta *et al.*, 1978) and lysozyme has been found in carcinoma cells (Tahara *et al.*, 1982). Enzymes such as acid phosphatase and alpha-naphthyl acetate esterase (ANAE), demonstrated by histochemical techniques, are also not specific and may be found in a variety of tumours including, in the case of ANAE, mammary carcinoma (Tubbs *et al.*, 1979). More recently, a variety of monoclonal antibodies with activity against MPS cells have been used in studies of tumour infiltrates (Steele *et al.*, 1985; Lwin *et al.*, 1985). Among the drawbacks associated with the use of such antibodies, however, is failure to detect all tissue macrophages as in the case of UCHM-l (Hogg *et al.*, 1984) used in the study of Lwin *et al.* (1985) or reactivity with non-MPS elements such as granulocytes as in the case of VEP-7 (Kraft *et al.*, 1981), the antibody used in the study of Steele *et al.* (1985).

We report here the results of a quantitative study of macrophages in benign and malignant breast disease using the mouse monoclonal antibody EBM/11 which has high cellular specificity for cells of the human MPS. A full account of this antibody, which was raised against human alveolar macrophages, is contained in Kelly *et al.* (1987) (see also Bliss *et al.*, 1984). Briefly, in an extensive tissue screen it showed reactivity against an epitope present in the cytoplasm of all members of the human mononuclear phagocyte system including peripheral blood monocytes, alveolar macrophages, Kupffer cells, splenic littoral cells, sinusoidal and germinal centre macrophages and also interdigitating reticulum cells in lymph nodes and microglial cells in the brain. Reactivity was also noted with macrophages in gut, dermis, liver, portal tracts, thyroid and kidney. There is also reactivity with other cells of bone marrow origin including osteoclasts, megakaryocytes and platelets (Athanasou *et al.*, 1986). However, both in tissue sections and in peripheral blood films, there is no reactivity with granulocytes or lymphocytes. In monocyte enriched preparations from peripheral blood virtually all cells stained with the exception of a few contaminating, morphologically recognizable lymphocytes. In the tissue screen, the only non myeloid element which showed weak reactivity was proximal renal tubule cells. No other epithelial cell stained. Endothelial cells did not stain. The antibody has been considered in the myeloid panel of the Third International Workshop on Human Leucocyte Differentiation Antigens, where it was assigned to Group 12, the mixed anti-macrophage group. Within this group, it is included in subgroup 4 which includes those antibodies with broad specificity (Hogg & Horton, 1987). Unlike many of the antibodies in this group EBM/11 shows strong reactivity with peripheral blood monocytes (Kelly *et al.*, 1987).

### Materials and methods

Thirty-four biopsies were studied. They were excision biopsies of clinically suspicious breast lesions which had been submitted for frozen section diagnosis. A portion of each biopsy was snap frozen in liquid nitrogen and stored at $-70°C$. Cryostat sections ($6\,\mu m$) were cut, air-dried and fixed in cold acetone for 30 min. Sections were incubated in tris-buffered saline (TBS), pH 7.4 with 20% pooled normal human serum before incubation with the primary antibody, EBM/11 (culture supernatant, 1:10 in TBS) for 30 min at 22°C. Negative controls were provided by substituting TBS for the primary antibody. Following washing in TBS for 10 min. a standard three stage immunoperoxidase technique was followed using diaminobenzidine (DAB) as a chromogen

Correspondence: P.M.A. Kelly.
Received 17 July 1987; and in revised form, 5 October 1987.

(Gatter *et al.*, 1984). In each case, frozen section diagnosis was confirmed by examining routine paraffin sections from the original frozen block which had been fixed in formal saline and embedded. In selected cases sections from the frozen paraffin blocks were stained using a polyclonal antibody to lysozyme (Hoechst: 1:200 in TBS). In cases of carcinoma, staging of the disease was determined following pathological examination of subsequent mastectomy and axillary lymph node specimens. In one case, segmental mastectomy was performed without axillary lymph node sampling. In this case, the staging was based on the assumption that the nodes were negative in accordance with the clinical findings. The tumours were graded histologically according to the criteria of Bloom & Richardson (1957). Pathological staging and histological grade were also combined into a prognostic index (PI) according to the simplified formula of Haybittle *et al.* (1982).

Macrophages, identified by positivity for EBM/11, were counted by projecting a high power microscope field onto a touch sensitive pad attached to a MOP-AMO3 (Kontron, German Federal Republic) image analyser. Three fields from the centre of the tumour were counted by each observer and the result was expressed as an average of the three. There was high concordance between the results. In the malignant cases, a count was also made of tumour cells in the same fields. U Test Counting was done independently by two observers. Each counted three fields from each section. In the tumour cases, the sections were from the main tumour mass. Fields were selected at random from the tumour sections and those counted consisted completely of tumour including stroma. Areas of necrosis were avoided. In malignant cases, a count was also made of tumour cells in the same fields. The second count was used as a check of consistency of the first. As three different random fields were chosen, it was also a test of how representative of the tumour the first sample was. There was high concordance between the results obtained by the two observers. This provided objective support for the subjective impression of little field-to-field variation in macrophage distribution within individual tumours (see **Results**).

The differences in macrophage count between the various subgroups were compared using the Mann–Whitney U Test.

### Results

Of the 34 cases studied, 17 were of benign disease [mean age 42.25 years (range: 37–57)]. All consisted of 'fibrocystic' disease, apart from one tubular adenoma and one case of gynaecomastia. The highest macrophage count in the benign group was in a case of sclerosing adenosis occurring in a pregnant woman. The remaining 17 cases were of malignant disease [mean age 56 years (41–70)]. The findings are summarised in Table I. Follow-up records were available for 14 of the carcinoma patients. The average period of follow-up was 24.1 months (range 4–33). No local recurrence or distant metastasis was recorded in that period.

Cells which were morphologically consistent with macrophages showed strong positivity on immunohistochemical staining with EBM/11. The corresponding cells in negative control sections remained unstained. Positive cells showed a uniform intensity of staining with little cell-to-cell variation. Mixed among the positively stained macrophages were other inflammatory cells such as lymphocytes, plasma cells and neutrophils. These were uniformly negative.

The macrophage counts for both groups are summarised in Table II. There is a significant difference in score between the benign and malignant groups, although the range for both is strikingly wide. In the malignant group macrophage counts showed no significant difference when stratified according to pathological stage (Table III) histological grade (Table IV) or prognostic index (Table V). In the absence of any recurrence, no attempt could be made to establish a relationship between macrophage count and this important determinant of disease activity, although such a relationship may become apparent with more prolonged follow-up.

Amongst the carcinomas, macrophages were found throughout the tumour both in the stroma and with tumour cell nests. Where sections included the junction between tumour and 'normal' breast there was no tendency for macrophages to collect at the tumour periphery. Many tumours contained very large numbers of macrophages which in some instances clearly outnumbered tumour cells (Table II). Within individual tumours the macrophage count was generally consistent throughout with only slight field-to-field variability. In no case was there staining of malignant cells by EBM/11. In some tumours, macrophages were more numerous in the stroma and appeared to infiltrate the tumour focally in a fashion resembling 'piecemeal necrosis' of the liver (Figure 1).

Although the sample from each case, three high power microscopic fields, was small, the concordance between the counts of the two observers indicated that the sample was adequate and representative.

In the cases where lysozyme was used as a marker for macrophages for comparison with EBM/11 many fewer positive cells were found overall. Those present were most numerous at the periphery of the tumour, in areas of necrosis and also within fibrous septa within the tumour, although some intraepithelial macrophages were also found (Figure 2).

**Table I** Summary of pathological stage and histological types and grade of 17 cases of breast carcinoma

| *Histological type* | *Clinico-pathological stage* I | II | Total | *Histological grade* I | II | III | Total |
|---|---|---|---|---|---|---|---|
| Lobular carcinoma in situ | 1 | – | 1 | 1 | – | – | 1 |
| Infiltrating lobular carcinoma | 1 | – | 1 | 1 | – | – | 1 |
| Infiltrating ductal carcinoma | 7 | 8 | 15 | 2 | 7 | 6 | 15 |
| Totals | 9 | 8 | 17 | 4 | 7 | 6 | 17 |

**Table II** Macrophage counts (per HPF) in benign and malignant breast tissue

| | *Mean*[a] | *(Range)* | *Mφ% of epithelial or tumour cells* | *(Range)* |
|---|---|---|---|---|
| Benign ($n=17$) | 54 | (15–183) | 25 | (2–120) |
| Malignant ($n=17$) | 99 | (21–185) | 53 | (7–139) |
| | $p<0.05$ | | | |

[a]Average of three high power fields.

### Discussion

This study demonstrates the presence, in significant numbers, of macrophages in breast tissue in both benign and malignant disease. Within both groups there is wide variation in numbers but there is, nevertheless, a significant excess in malignant tumours. The highest count, by far, in the benign

**Table III** Macrophage counts in malignant breast tumours according to pathological stage at presentation

| *Stage* | *Mean* | *Range* |
|---|---|---|
| I ($n=9$) | 96 | 21–173 |
| II ($n=8$) | 102 | 50–185 |

Not significant.

**Table IV** Macrophage counts in malignant breast tumours according to histological grade

| *Grade* | *Mean* | *Range* |
|---|---|---|
| I ($n=4$) | 74 | 46– 92 |
| II ($n=8$) | 97 | 21–185 |
| III ($n=5$) | 123 | 75–173 |

I *vs.* II; II *vs.* III; I *vs.* III; 1+II *vs.* III; I *vs.* II+III: all NS.

**Table V** Macrophage count according to prognostic index

| *PI* | *Mean* | *Range* |
|---|---|---|
| A ($n=5$) | 91 | 21–185 |
| B ($n=9$) | 115 | 50–173 |
| C ($n=2$) | 134 | 110–157 |

$A \leqq 3.4$ $C > 5.4$

A *vs.* B; B *vs.* C; A *vs.* C; A+B *vs.* C; A *vs.* B+C: all NS.

**Figure 1** A cryostat section of infiltrating ductal carcinoma which includes the edge of the tumour. Abundant EBM/11$^+$ macrophages are present throughout the tumour and are seen to extend into it from the periphery in a fashion reminiscent of 'piecemeal necrosis' (arrow) (Immunoperoxidase x 32).

group was in a biopsy from a pregnant woman which showed sclerosing adenosis. In the malignant group, there is no correlation between pathological stage, histological grade or prognostic index and macrophage count, although the numbers in each group are small. In the period of follow-up, no recurrence was documented although the average follow-up, at just over two years is relatively short in the natural history of this disease.

The finding of large numbers of macrophages within benign and malignant breast tissue is in accordance with recent observations by Lwin *et al.* (1985), Ferguson (1985), and Steele *et al.* (1985). We also observed a significant number of intraepithelial macrophages in benign breast tissue as well as within tumour epithelium (Lwin *et al.*, 1985). We did not quantitate separately intraepithelial and stromal macrophages in tumours as, especially in the diffusely infiltrative or solid tumours, the distinction would have been arbitrary. However, on subjective assessment, in some tumours the macrophages were predominantly stromal whereas, in others the distribution was more diffuse. There is a disparity between our findings and those of workers who have studied macrophage infiltration of mammary carcinoma using lysozyme as a marker (Nash, 1981; Tanaka *et al.*, 1986). These studies showed the presence of small numbers of macrophages associated with the tumours which were localized mainly at the periphery. In the cases which we studied using lysozyme, our findings were similar to those reported but were in marked contrast to those with EBM/11. This is not attributable to differences in fixation and processing as, in a separate study, we have found that routine fixation in formalin and processing through to paraffin does not affect reactivity with the polyclonal antibody which was used in this study (Kelly, in preparation). The difference in macrophage numbers found using the two different antibodies suggests that many macrophages in tumours are either depleted of lysozyme because of rapid turnover or that some are no longer synthesizing it in immunohistochemically-detectable quantities. The persistence of lysozyme containing macrophages in some parts of tumours, i.e. in areas of necrosis and at the periphery, further suggests a functional dichotomy in that many fewer lysozyme positive macrophages are found in intimate contact with tumour cells or in tumour stroma. We have noted a similar depletion of lysozyme containing Kupffer cells in alcoholic liver biopsies although there is no reduction in absolute Kupffer cell numbers in these livers using EBM/11 as a marker (Kelly, in preparation). Clearly, therefore, lysozyme is not a reliable marker for use alone in quantitative histological studies of macrophages although when used in parallel with a marker such as EBM/11, it may give a useful insight into the functional state of MPS cells in various disease states.

The role of macrophages in tumours in general and specifically in breast tumours is not fully understood. It is clear that this role is not a simple one and that tumour associated macrophages (TAM) are functionally heterogeneous (McBride, 1986). The balance of evidence from experimental studies supports the notion that macrophages do have a role in limiting metastasis and that this function can be carried out without help from other immuno-

**Figure 2** A similar field to **Figure 1** showing macrophages stained for lysozyme. There are many fewer positive cells. Those present are most numerous at the edge of the tumour or in fibrous septa with only scattered intra-tumoral macrophages (Immunoperoxidase x 32).

competent cells (Fidler & Poste, 1982), although *in vivo* the invariable association of lymphocytes with macrophages in tumours cannot be ignored. The finding in this study of large numbers of macrophages within malignant breast tumours including the tumour epithelium itself, the so called 'neoplastic compartment' (Whitwell *et al.*, 1984), is not inconsistent with the concept of anti-tumour activity of TAM although we recognise the dangers of making inferences about function from static morphological studies such as this.

This study also demonstrates the usefulness of a monoclonal antibody such as EBM/11 as a marker for macrophages in immunohistochemical investigations of malignant tumours. Its use has highlighted the presence of a large component of MPS cells within malignant breast tumours. The number of such cells was not apparent and certainly not quantifiable on conventional staining and would have been seriously underestimated if a marker such as lysozyme had been used.

We thank Miss Lesley Watts for typing the manuscript. PMAK holds a travelling studentship from the National University of Ireland.

### References

ATHANASOU, N.A., HERYET, A., QUINN, J., GATTER, K.C., MASON, D.Y. & McGEE, J. O'D. (1986). Osteoclasts contain macrophage and megakaryocyte antigens. *J. Path*, **150**, 239.

BENITEZ-BIBIESCA, L. & FRERE-HORTA, R. (1978). Immunofluorescent localization of alpha-1 antitrypsin in human polymorphonuclear leukocytes. *Life Sci.*, **21**, 99.

BLISS, E., NAIEM, M., BURNS, J., BELL, K. & McGEE, J.O'D. (1984). Quantification of macrophages in human breast cancer using monoclonal antibody (EBM/11) to human macrophages. *J. Pathol.*, **143**, A6 (*Abstract*).

BLOOM, H.J.G. & RICHARDSON, W.W. (1957). Histological grading and prognosis in breast cancer. *B. J. Cancer*, **11**, 359.

ECCLES, S.A. & ALEXANDER, P. (1974). The macrophage content of tumours in relation to metastatic spread and host immune reaction. *Nature*, **250**, 667.

FERGUSON, D.J.P. (1985). Intraepithelial lymphocytes and macrophages in the normal breast. *Virchows Arch.* (Pathol. Anat.), **407**, 369.

FIDLER, I.J. & POSTE, G. (1982). Macrophages and cancer metastasis. In *Macrophages and Natural Killer Cells*. Adv. exptl. med. biol., **155**, Norman, S.J. & Sorkin, E. (eds) p. 65. Plenum Press: New York.

GATTER, K.C., FALINI, B. & MASON, D.Y. (1984). The use of monoclonal antibodies in histopathological diagnosis. In *Recent Advances in Histopathology*, No. 12. Anthony, P.P. & MacSween, R.N.M. (eds) p. 35. Churchill Livingstone: Edinburgh.

HAMLIN, I.M.E. (1968). Possible host resistance in carcinoma of the breast: a histological study. *Br. J. Cancer*, **22**, 383.

HAYBITTLE, J.L., BLAMEY, A.W., ELSTON, C.W. & 5 others (1982). A prognostic index in primary breast cancer. *Br. J. Cancer*, **45**, 361.

HOGG, N., MacDONALD, S., SLUSARENKO, M. & BEVERLEY, P.C.L. (1984) Monoclonal antibodies specific for human monocytes, granulocytes and endothelium. *Immunology*, **53**, 753.

HOGG, N. & HORTON, M.A. (1987). Myeloid antigens: new and previously defined clusters. In *Leucotype Typing III*, McMichael, A. (ed) p. 576. Oxford.

ISAACSON, P., JONES, D.B., MILLWARD-SADLER, G.H., JUDD, M.A. & PAYNE, S. (1981). Alpha-l antitrypsin in human macrophages. *J. Clin. Pathol.*, **34**, 982.

KELLY, P.M.A., BLISS, E., MORTON, J.A., BURNS, J. & McGEE, J.O'D. A monoclonal antibody, EBM/11, with high cellular specificity for human macrophages. *J. Clin. Pathol.*, (in Press).

KRAFT, D., RUMPOLD, K., STEINER, R. & 4 others (1981). Evidence against a myeloid nature of human large granular lymphocytes (LGL). In *Mechanisms of Lymphocyte Activation*, Resch & Kirchner (eds) p. 279. Elsevier/North-Holland Biomedical Press: Amsterdam.

LAUDER, I., AHERNE, W., STEWART, J. & SAINSURY, R. (1977). Macrophage infiltration of breast tumours: a prospective study. *J. Clin. Pathol.*, **30**, 563.

LWIN, K.Y. ZUCCARINI, O., SLOANE, J.P. & BEVERLEY, P.C.L. (1985). An immunohistological study of leucocyte localization in benign and malignant breast tissue. *Int. J. Cancer*, **36**, 433.

McBRIDE, W.H. (1986). Phenotype and functions of intratumoral macrophages. *Biochim. Biophys. Acta*, **865**, 27.

MASON, D.Y. & TAYLOR, C.R. (1975). The distribution of muramidase (lysozyme) in human tissue. *J. Clin. Pathol.*, **28**, 1488.

NASH, J., PRICE, J. & TARIN, D. (1979). Macrophage content and colony-forming potential in mouse mammary carcinoma. *Br. J. Cancer*, **39**, 478.

NASH, J. (1982). Macrophages in human tumours: an immunohistochemical study. *J. Pathol*, **136**, 73.

SONE, S., TACHIBANA, K., ISHII, K., OGAWARA, M. & TSUBARA, E. (1984). Production of a tumour cytolytic factor(s) by activated human alveolar macrophages and its action. *Cancer Res.*, **44**, 646.

SONE, S., LOPEZ-BERESTEIN, G. & FIDLER, I. (1985). Kinetics and function of tumor cytotoxic factor(s) produced by human blood monocytes activated to its tumoricidal state. *J. Natl. Cancer Inst.*, **74**, 583.

STEELE, R.J.C., BROWN, M. & EREMIN, O. (1985). Characterization of macrophages infiltrating human mammary carcinomas. *Br. J. Cancer*, **51**, 135.

TAHARA, E., ITO, H., SHIMATNOTO, F., IWAMOTO, T., NAKAGAMI, K. & NIIMOTO, H. (1982). Lysozyme in human gastric carcinoma: a retrospective immunohistochemical study. *Histopathology*, **6**, 409.

TANAKA, H., SHIMODA, T., UCHIDA, K., SUZUKI, T. & ISHIKAWA, E. (1986). Immunohistochemical study of the distribution and significance of mononuclear cells in human breast carcinoma. *Acta. Pathol. Jpn.*, **36**, 1455.

TUBBS, R.R., SAVAGE, R.A., CRABTREE, R.H., SEBEK, B.A., VALENZUELA, A. & BENJAMIN, S.P. (1979). Expression of monocytic-histiocytic cytochemical markers in epithelial neoplasia. *Am. J. Clin. Pathol.*, **72**, 789.

WHITWELL, H.L., HUGHES, H.P.A., MOORE, M. & AHMED, A. (1984). Expression of major histocompatibility antigens and leucocyte infiltration in benign and malignant human breast disease. *Br. J. Cancer*, **49**, 161.

WOOD, G.H. & GILLESPIE, G.Y. (1975). Studies on the role of macrophages in regulation of growth and metastasis of murine chemically induced fibrosarcomas. *Int. J. Cancer*, **16**, 1022.